# A Thematic Analysis of Key Informant Interviews on the Implementation Experience of the HEARTS Hypertension Program in Nigeria

**DOI:** 10.5334/gh.1566

**Published:** 2026-06-19

**Authors:** Muhammad Jami Husain, Malau Mangai Toma, Sunday Victor Eze, Kufor Osi, Nanlop Ogbureke, Okeoma Erojikwe, Deliana Kostova, Andrew E. Moran, Bolanle F. Banigbe, Emmanuel Ndenor Sambo

**Affiliations:** 1 Division of Global Health Protection, Centers for Disease Control and Prevention, Atlanta, GA, USA; 2 Non-Communicable Diseases Division, Department of Public Health, Federal Ministry of Health and Social Welfare, Abuja, Nigeria; 3 Resolve to Save Lives, Abuja, Nigeria; 4 Resolve to Save Lives, New York, NY, USA; 5 Columbia University, New York, NY, USA; 6 UKS Consulting, Abuja, Nigeria

**Keywords:** Nigeria Hypertension Control Initiative (NHCI), HEARTS technical package, key informant interviews, primary care, task shifting

## Abstract

The Nigeria Hypertension Control Initiative (NHCI) launched a national treatment program in 2020, guided by the HEARTS technical package for cardiovascular disease prevention in primary care. This study presents a thematic analysis of key informant interviews with primary care experts from Abuja, Kano, and Ogun, exploring NHCI implementation experiences. Using a structured qualitative approach, we analyzed responses across four domains: service delivery, medication procurement and availability, task shifting, and broader program aspects. Findings highlight improvements in service delivery, medication access, task shifting, and health-seeking behavior. However, challenges persist, including incomplete training, user fees, administrative burdens, and logistical barriers such as transportation costs and stockouts. Despite these issues, satisfaction with the standardized treatment protocol was high. Task shifting was well-received, easing pressure on senior staff but underscoring the need for continued training. Recommendations include integrating diabetes care, decentralizing medication sourcing, and enhancing training to strengthen NHCI’s efficiency and sustainability in resource-limited settings.

## Introduction

Nigeria faces a growing burden of hypertension, with adult prevalence rising from 8.6% in 1995 to 32.5% in 2020, amid major health system challenges including poor integration of non-communicable disease care, frequent medication shortages, limited insurance coverage, and inadequate workforce capacity (1,2,3). In response, the Nigeria Hypertension Control Initiative (NHCI) launched a national program in 2020 based on the HEARTS technical package, emphasizing systematic screening, lifestyle counseling, and standardized treatment protocols delivered through team-based primary care (4,5). This report offers a thematic analysis of key informant interviews on implementing the HEARTS approach to hypertension management, utilizing qualitative responses from primary care facility experts in Kano and Ogun states and the Abuja Federal Capital Territory (FCT). The aim is to uncover key themes related to service delivery, medication procurement and availability, task shifting, and other aspects of the program to provide insights into the strengths and challenges of the HEARTS approach and offer actionable recommendations to improve the program’s effectiveness.

## Method

### Study setting

The NCHI hypertension management program serves as a model for HEARTS implementation, offering structured guidance on lifestyle counseling, standardized treatment, essential medicines, team-based care, and monitoring. It aims to expand hypertension care through a team-based approach that empowers nurses and community health workers. Nigeria’s HEARTS protocol follows a structured regimen, starting with amlodipine 5 mg daily and progressing to combinations with losartan and hydrochlorothiazide as needed. Generic medications are available at public facilities to ensure access. The initiative has been piloted in 104 primary health centers in Kano and Ogun, enrolling about 35,000 patients, with 60 additional clinics in Abuja FCT participating in a University of Abuja hypertension outcomes study.

### Key informant interviews and thematic analysis

The interview guide was developed specifically for this study by the research team, was structured around four HEARTS/NHCI implementation domains—service delivery, medication procurement and availability, task shifting, and broader program operations—and was not adapted from a pre-existing validated instrument. Eleven open-ended questions were posed across these four domains (Table 1). The study followed the Standards for Reporting Qualitative Research (SRQR) framework (6).

**Table 1:** List of interview questions.


DOMAIN	QUESTION

**Service delivery**	- In your opinion, what are the main differences between the HEARTS approach and usual care for hypertension/diabetes? - What are some main challenges with the HEARTS approach? Examples include training issues, adoption of the stepwise standardized protocol by providers, shifting of tasks between providers, etc. - Can you identify any inefficiencies in service delivery with the HEARTS program? Can you provide some examples? (e.g. patients wait time, providers prescribing medications outside of the HEARTS protocol, etc.)

**Medication procurement and availability**	- Did the facility encounter any difficulty in securing an adequate supply of medications? - What is the procurement source for the medications used in the HEARTS and/or usual care protocols for hypertension and/or diabetes? - Did the facility face any challenges with the medications supply chain, such as late delivery, poor quality, unstable supply, or other issues? Can you provide examples? - Were healthcare providers satisfied with the HEARTS protocol for hypertension? If not, what were some reasons?

**Task shifting**	- What is healthcare providers’ perception of task-shifting, such as when nurses prescribe hypertension medications under the HEARTS protocol? - Did you identify any inefficiencies/challenges with task-shifting with the HEARTS program? - What activities are most likely to be delegated from doctors to nurses?

**Miscellaneous**	- What other aspects of the HEARTS program would you like to comment on, including positive and/or challenging aspects not discussed above?


Fifteen key informants were purposively selected, one from each NHCI/HEARTS-participating primary care facility: five in the Abuja Federal Capital Territory (FCT), five in Kano State, and five in Ogun State. At each facility, the respondent was the Health Facility Officer-in-Charge or the designated hypertension/non-communicable disease focal person. These respondents had direct frontline management or clinical coordination responsibilities for HEARTS/NHCI implementation in their facilities and were therefore positioned to comment on service delivery, medication procurement and availability, task shifting, and broader program operations. Where relevant, respondents corroborated answers by consulting other facility staff responsible for specific operational tasks, such as drug supply or data reporting. Interviews were conducted face-to-face by the principal investigator between February and April 2024 and lasted approximately one hour each.

Following Naeem et al. (2023) (7), we conducted a structured, multi-step thematic analysis. The principal investigator (PI) conducted in-person interviews with key informants, each lasting about an hour. Responses were manually transcribed into Microsoft Excel and organized by facility and question. During initial coding, the PI and lead author independently reviewed transcripts, highlighted key excerpts, and assigned concise response codes. These were grouped into broader themes by identifying recurring concepts, with Microsoft Copilot assisting in generating an initial list. All authors collaboratively refined and named the themes to ensure clarity and alignment with the data. Final themes and codes were then applied to interpret implementation experiences across facilities. The counts presented in Table 2 reflect the number of key informants whose responses contributed to each code; codes are not mutually exclusive, and counts are descriptive within this purposive sample rather than prevalence estimates.

**Table 2:** Findings of the thematic analysis from the key informant survey responses.


DOMAIN	QUESTION THEME	RESPONSE THEME	RESPONSE CODES	ABUJA n = 5	KANO n = 5	OGUN n = 5	TOTAL (N = 15)

**Service delivery**	Main differences between the HEARTS approach and usual care for hypertension/diabetes	Enhanced Hypertension Management	Improved capacity for hypertension management	4/5	4/5	3/5	11/15

			Deliberate focus on hypertension patients		4/5		4/15

			Adherence to protocol	2/5	2/5	1/5	5/15

		Task Shifting and Service Delivery	Task shifting and its impact on service delivery	2/5			2/15

			Improved health-seeking behavior	1/5			1/15

			Increased awareness of hypertension		1/5		1/15

			Improved capacity for detection and management		4/5		4/15

			Improved capacity for tracking loss to follow-up (LTFU) patients		4/5		4/15

		Access to Medications	Improved access to quality-assured, relatively cheap medicines	4/5	2/5	2/5	8/15

	Main challenges with the HEARTS approach	Training and Implementation Challenges	Incomplete training of health workers	1/5			1/15

			Data burden on health workers			2/5	2/15

		Financial and Medication Issues:	Introduction of user fees leading to dropouts and LTFU	4/5			4/15

			Side effects of medications	1/5			1/15

			No challenges reported		5/5	3/5	8/15

	Inefficiencies in service delivery with the HEARTS program	Resource Utilization and Training:	Underutilization of health workers	1/5			1/15

			Need for periodic retraining			1/5	1/15

		Equipment and Maintenance Issues:	Delays in equipment maintenance			1/5	1/15

**Medication procurement and availability**	Difficulty in securing an adequate supply of medications	Logistical Challenges:	Difficulty in staff making trips for refills	1/5			1/15

			No difficulty reported	3/5	4/5	3/5	10/15

	Procurement source for medications	Procurement Sources	Abuja University Teaching Hospital	5/5			5/15

			Drug Management and Consumable Supply Agency (DMCSA)		3/5		3/15

			From the state			4/5	4/15

	Challenges with the medication supply chain	Supply Chain Issues	Cost of transportation	1/5			1/15

			Stockouts			1/5	1/15

			No challenges reported	3/5	5/5	4/5	12/15

	Satisfaction with HEARTS protocol	Provider Satisfaction	Satisfaction with the protocol	5/5	2/5	5/5	12/15

**Task shifting**	Perception of task shifting	Improved Service Delivery	Reduced burden for senior cadre	1/5		1/5	2/15

			Improved service delivery	4/5	4/5	5/5	13/15

			Confidence boost for lower cadres	2/5			2/15

	Inefficiencies/challenges with task shifting	Training Needs	Need for training all health workers	1/5			1/15

			No inefficiencies identified	3/5	5/5	4/5	12/15

	Activities delegated from doctors to nurses	Delegation of Tasks	All tasks can be delegated	1/5	2/5	4/5	7/15

			Enrollment counseling, screening, and treatment	3/5	3/5		6/15

**Miscellaneous**	Other aspects of HEARTS program	Program Expansion and Integration	Integration of diabetes care	3/5			3/15

			Decentralizing medicine sourcing	1/5			1/15

		Affordability and Training	Affordability of drugs	1/5	2/5	2/5	5/15

			Need for more training			1/5	1/15

		Support and Maintenance	Excitement about DHIS 2 deployment		1/5		1/15

			Support for equipment maintenance			1/5	1/15

			Strengthening medicine supply/availability			1/5	1/15


*Note:* Counts indicate the number of key informants/facilities in each state whose individual response explicitly or substantively reflected the response code. Denominators are five per state. Counts are not mutually exclusive because a single response could contribute to more than one code. Blank/non-responsive cells indicate that the code was not reflected in any response from that state.

## Key Findings

Table 2 presents the findings of the thematic analysis based on interviewees’ responses to 11 questions covering four program domains. The analysis generated 40 response codes grouped under 17 response themes. For each response code, counts are presented as n/5 by location and n/15 overall, indicating the number of key informants whose responses contributed to that code. Codes are not mutually exclusive; a single response could contribute to more than one code. Because participants were purposively selected, these counts are intended to show distribution within this sample and should not be interpreted as prevalence estimates.

### Service delivery

Respondents reported improved hypertension service delivery under the HEARTS protocol compared to usual care. In Abuja, four of five respondents noted enhanced competence and capacity in hypertension management, and four of five reported improved access to quality-assured, affordable medicines. In Kano, improvements in detection and management capacity were reported by four of five respondents, with four of five also noting improved capacity for tracking loss to follow-up patients. In Ogun, three of five respondents noted improved hypertension management capacity using the standardized HEARTS protocol. Despite these gains, challenges remain. In Abuja, four of five facilities reported patient dropouts after user fees were introduced following an initial period of free medication. One facility cited inefficiencies due to underutilized health workers who had not been trained or empowered to provide services. Respondents also emphasized the need for periodic retraining and timely equipment maintenance. In Ogun, two facilities reported increased data collection burdens on healthcare workers, potentially affecting service efficiency.

### Medication procurement and availability

The medications supply chain presented varying challenges across different states. Three of five Abuja respondents reported no difficulty securing medication supply, with one Abuja facility flagging staffing constraints that limited refill trips and another noting initial-period stockouts. All Kano respondents and four of five Ogun respondents reported no significant supply difficulties. One Ogun facility reported a current losartan stockout. Despite these site-specific issues, there was a general sense of satisfaction with the HEARTS protocol across all three states (12 of 15 facilities expressed satisfaction). The consistent application of the protocol contributed to the perceived improved healthcare outcomes and positive feedback from the healthcare providers, underscoring the importance of reliable medication supply chains.

### Task shifting

Task shifting was widely seen as a positive development within the HEARTS program. Improved service delivery as a result of task shifting was reported across nearly all sites (13 of 15 facilities). It enabled more efficient task allocation, allowing nurses and community health workers to take on responsibilities typically handled by doctors, such as enrollment counseling, screening, and treatment. Two of five Abuja respondents specifically noted a confidence boost among lower-cadre health workers as an additional benefit. However, the need for comprehensive training was highlighted by respondents in one Abuja facility, underscoring the importance of equipping staff with the skills and knowledge to carry out these delegated tasks. Thorough training is essential to maintain high standards of care and maximize the benefits of task shifting.

### Program integration and support needs

Several additional aspects were highlighted that could further enhance the HEARTS program’s effectiveness. Integrating diabetes care into Nigeria’s HEARTS framework was suggested by respondents from three of five health facilities in Abuja to support a more holistic approach to chronic disease management. To address logistical challenges and improve medication affordability, decentralizing sourcing was recommended by one Abuja facility. Medication access and affordability issues were also noted by one facility in Abuja, two in Kano, and two in Ogun. In Ogun, there was also a call for increased training and support for health workers at one facility, and equipment maintenance at another—emphasizing the need for ongoing investment in healthcare infrastructure and workforce development.

## Discussion

This study highlights improvements in hypertension management through the HEARTS approach, particularly via task shifting, which eased the burden on senior staff and improved patient care. However, challenges persist, including training gaps, data collection burdens, and supply chain inefficiencies. These findings align with existing literature on the benefits of standardized protocols and task shifting, while also echoing common challenges in training and logistics (8,9,10).

Several actions may enhance NHCI HEARTS implementation: strengthening training programs for health workers; improving the supply chain to prevent medication stockouts; and reducing administrative burden. Additionally, full implementation of Nigeria’s 2022 National Health Insurance Act could help address financial barriers and improve retention (11).

Key Informant Interviews (KIIs) provide valuable qualitative insights but have notable limitations. They are prone to bias, errors, and misinterpretation due to reliance on subjective perspectives. Selection bias may occur if informants share similar views, limiting diversity of input. Detailed individual-level characteristics of key informants, such as cadre, sex, age, and years of experience, were not collected; therefore, the informant profile is limited to their facility-level program roles and site characteristics. Interviewing only one expert per facility may not capture the full range of experiences. Time constraints can restrict deeper exploration of complex topics, and interviewer style may influence responses.

Facility-level clinical or administrative outcome data, such as blood pressure control, patient retention, loss to follow-up, and medication stockout rates, were not collected as part of this qualitative protocol. Consequently, the themes reported here should be interpreted as key informants’ facility-level perceptions of HEARTS/NHCI implementation experience rather than as findings formally triangulated with routine service data. Future research should link qualitative implementation experiences with facility-level clinical and administrative metrics to more rigorously assess the validity, generalizability, and operational implications of the themes identified here.

Despite these limitations, the HEARTS program shows strong potential for scale-up due to its standardized treatment protocols and adaptable, team-based care model. The study’s recommendations (enhancing training, refining supply chains, and streamlining administrative processes) aim to improve operational efficiency and sustainability.

## Use of Generative AI Tool

Microsoft Copilot was used during one of the methodological steps to assist in generating an initial list of themes from coded responses. Each theme was subsequently reviewed, defined, named, and validated by all authors to ensure it accurately reflected the underlying data. The authors take full responsibility for the integrity, accuracy, and originality of the manuscript, and affirm that the content represents their own ideas, knowledge, and intellectual contributions.

## Data Availability

The datasets used and/or analyzed during the current study are reported in the manuscript and available from the corresponding author on reasonable request.
